# Polisomnographic findings on children with laryngopathies

**DOI:** 10.1016/S1808-8694(15)30054-9

**Published:** 2015-10-19

**Authors:** Michele Themis Moraes Gonçalves, Juliana Sato, Melissa A.G. Avelino, Gilberto U. Pizarro, Gustavo A. Moreira, Márcia Pradella Hallinan, Reginaldo R. Fujita, Luc Louis Maurice Weckx

**Affiliations:** a MD, Otorhinolaryngology Resident - UNIFESP.; b MD, Otorhinolaryngology Resident - UNIFESP.; c MD, MS, Postgraduate student (PhD) in Otorhinolaryngology UNIFESP-EPM.; d MD, Postgraduate student (MS) in Otorhinolaryngology -UNIFESP-EPM.; e MD, MS in Pediatrics - UNIFESP-EPM, Professor of Sleep Medicine and Biology UNIFESP-EPM.; f MD, MS in Pediatrics - UNIFESP-EPM Professor of Sleep Medicine and Biology UNIFESP-EPM.; g PhD in Otorhinolaryngology - UNIFESP-EPM. Head of Pediatric Otorhinolaryngology -UNIFESP-EPM.; h PhD in Otorhinolaryngology - UNIFESP-EPM. Head of Otorhinolaryngology -UNIFESP-EPM. UNIFESP-EPM.

**Keywords:** Polysomnography, Larynx, Apnea, Children

## Abstract

Polysomnography is the goldstandard exam for child OSAS. When possible, polysomnography clearly distinguishes between those with isolated primary snoring and patients with sleep apnea (obstructive, central and mixed). The most common cause of OSAS in childhood is adenotonsillar hypertrophy. Laryngomalacia is the most common cause of stridor in childhood, though its physiopathology remains unknown. Among the most prominent theories are immaturity of the cartilaginous framework of the larynx and/or neuromuscular immaturity. **Objective:** Our proposal was to describe polysomnographic findings in children with laryngomalacia or other isolated laryngeal alterations, that is, without other alterations in the upper airways. **Methods:** The sample included 29 children with exclusively laryngeal alterations. All of them underwent an otorhinolaryngological exam, nasofibrolaryngoscopy and polysomnography. Information was recorded concerning age, nasofibrolaryngoscopy and polysomnography. For analysis, the children were divided into two groups: those with laryngomalacia and those with other laryngeal diseases. **Results:** Among the 18 children with a diagnosis of laryngomalacia, 18 had central breathing events, knowing that the majority had showed dessaturation of oxihemoglobin and bradicardia. In this same group, 3 children had obstrutives events. On the other hand, 11 children with other laryngeal alterations showed no predominance of one type or another of apnea. Of these, 4 had central type breathing events and 2 obstructive type. **Conclusion:** The majority of patients with laryngomalacia showed a central type apnea. Patients with various laryngeal diseases did not present a predominant type of apnea.

## INTRODUCTION

Snoring and mouth breathing are common complaints in childhood, with incidence rates varying from 3 to 26%. In the pediatric population, we have 1 to 3 % of patients with Sleep Obstruction Apnea Syndrome (SOAS), and snoring is the most common symptom.

Snoring is what we call the vibratory sound produced in the nasopharynx during inspiration. Primary snoring is not related to apnea, hypoventilation, hypoxia, hypercarbia, restless sleep or excessive daily sleepiness.

SOAS in children is characterized by partial or complete obstruction of the upper airways during sleep, usually followed by a drop in oxyhemoglobin saturation and/or hypercarbia. The first series of SOAS cases in children was published in 1976 by Guilleminault et al^3^. SOAS in children is different from its adult counterpart. In children the disease comprises episodes of isolated obstructive hypoventilation, apnea associated to obstructive hypoventilation, and plain obstructive apnea. In other words, the sleep obstructive respiratory disorders are called SOAS even when there are no apnea episodes.

By definition, apnea is the lack of airflow by either the nose or the mouth. It may be caused by an interruption in respiratory movements (central-type apnea) or by a collapse of the upper airways (obstructive-type) apnea. When we have central and obstructive apnea occurring simultaneously in children, in order to be considered relevant, they should last more than 2 respiratory cycles and are not always associated to hypoxemia^4^. Short duration central apnea (less than 15 seconds) is a common finding in the sleep of normal newborn children^5^.

Insofar there is no criterion that defines an acceptable number of central apnea episodes. Notwithstanding, if associated to bradicardia or cyanosis, regardless of its duration, it is considered significant. Heart-related, blood-related, infectious, metabolic, neurological, pulmonary, gastrointestinal and neuromuscular causes are mentioned for central apnea, although most of the times they are not identified.

Hypoventilation means a drop in pulmonary ventilation below the minimum necessary to sustain normal oxygen saturation. Just like apnea, it may also happen from non-obstructive causes (lower central respiratory stimulus, neuromuscular abnormalities or pulmonary restrictive diseases) or obstructive (partial obstruction of upper airways causing inadequate pulmonary ventilation) causes.

Contrary to what happens in adults, in whom the major SOAS symptom is daily sleepiness, children are most often taken to the doctor, because of snoring or respiratory difficulty during sleep. Night time awakenings, chronic mouth breathing, excessive daily sleepiness, psychomotor restlessness, excessive movements during sleep are also frequent. Children with SOAS may have low school performance, disciplinary problems, attention deficits, mood alterations (irritability, aggressiveness), swallowing disorders and voice changes^30^, ^31^. Besides all of these, there may be important growth deficits because of alterations in GH secretion during sleep^6^, ^7^, ^8^. Other hypothesis, still not confirmed, is that weight and height deficit may be secondary to greater energy expenditure during a more laborious breathing or due to less calorie ingestion caused by poor appetite or disphagia^9^, ^10^, ^11^, ^12^. Severe cases may be associated to cor pulmonale and death.

Pediatric SOAS is not fully understood. We know that there is no single mechanism responsible, and that it is very likely that pediatric SOAS with adenotonsillar hypertrophy be different from pediatric SOAS with craniofacial or neurological syndromes.

The otorhinolaryngological exam is very important and starts by facial inspection, looking for retrognatism or hypognatism. After that we assess tongue size and its relationship with the dental arch, presence of tonsil hypertrophy, high palate and cross bite. In the nose, we look for inferior turbinates hypertrophy or cyanosis, septal deviations, secretions, polyps or tumors. Nasofibroscopy is necessary and very important because it may define, in children, the sites of upper airway narrowing, adenoid-choana ratio, tongue base and oropharynx with the pharyngeal posterior wall, side to side tonsil collapse, signs of gastroesophageal reflux or glottic and subglotic alterations.

As it happens with adult patients, the gold standard tests for childhood SOAS is the polysomnography, in order to assess both the severity and the therapy mode to be followed. It is a test that requires skilled professionals in dealing with children, and very important is active parent participation. When carried out, polysomnography clearly separates those bearers of primary snoring from sleep apnea patients (obstructive, central and mixed).

Children SOAS diagnostic criteria were only recently defined. In children, one or more obstructive-type apnea episodes per hour and/or obstructive-type hypoventilation, characterized by peak CO2 exhaled (ETCO2) = 53 mmHg, ETCO2 > 50 mmHg in more than 10% of total sleep time or ETCO2 > 45 mmHg in more than 60% of total sleep time, are considered to be abnormal. HAI greater than 10 is considered very severe^13^. The polysomnography test of primary snorers have hypopnea/apnea index (HAI) lower than 1 episode per hour, oxygen saturation average during sleep greater than 90% and ETCO2 peak < 53 mmHg or ETCO2 peak = 50 mmHg in less than 10% of total sleep time^14^. As to the assessment of sleep efficacy, it is considered abnormal when it is lower or equal to 85%^4^.

The most common cause of SOAS in children is adenotonsillar hypertrophy6, and chronic hypertrophic allergic rhinitis makes it even worse. It is also very important to check other anatomical causes (nasal septum deviation, nasal polyps, choanal stenosis, tongue hypertrophy, micrognatia, laryngomalacia, laryngeal diaphragm), congenital and syndrome diseases (Apert, Cri du Chat, Crouzon, Down, Pierre Robin, Treacher-Collins syndromes; mucopolysacaridosis, cystic fibrosis, Arnold-Chiari, brain palsy) among others (myopathy, neuromuscular disease, neoplasia, family issues, gastroesophageal reflux).

Laryngomalacia is the most common cause of children stridor^15^, although its prevalence is unknown. Literature data report incidence rates varying from 19.4 to 75%^16^, ^17^. Stridor usually appears after the first weeks of life and may remain until 18-24 months. Stridor may increase in restlessness, feeding or supine position. It happens because of supraglottic collapse secondary to excess mucosal tissue in the larynx posterior wall, shortening of the aryepiglottic fold or epiglottis cartilage falling over the larynx lumen. Its cause is still debated. There are many theories proposed to explain its physiopathology, such as disorders in the cartilaginous framework of the larynx and trachea, causing a greater laxity in supraglottic structures, anatomical alterations and neuromuscular immaturity^18^, ^19^, ^20^, ^21^.

Back in 1897, Sutherland & Lack^22^ proposed an anatomical theory after studying 18 cases of congenital laryngeal obstruction, in which they concluded that the disorder was associated to the immaturity of the children cartilaginous tissue. Prescott^23^ added to this hypothesis by studying 40 patients with laryngomalacia and noticed that all of them had short aryepiglottic folds and 30% had neuromuscular disorders.

Thompson and Turner^24^ demonstrated that a passive medial supraglottic prolapse could be induced from the denervation of children larynxes. This study, together with others such as the Perón et al.^25^ and Wiggs and Dinardo^26^, established the neurological hypothesis. The neurological theory explains the association between laryngomalacia and other neurological disorders^22^, ^27^. Belmont and Grundfast^28^ found 80% of LFR, 13% of hypotonia and 10% of central apnea in 30 children with laryngomalacia.

The diagnosis of laryngomalacia is achieved through flexible laryngoscopy during spontaneous breathing. It is usually of spontaneous resolution. Literature reports indicate that severe laryngomalacia is uncommon to the point of causing cyanosis, feeding difficulties, cor pulmonale, apnea or growth impairment. When it happens, patients with severe laryngomalacia are usually treated by tracheostomy, notwithstanding there are a number of other surgical treatments (aryepiglottic fold resection, epiglotoplasty and supraglotoplasty).

## OBJETIVE

Our goal was to describe polysomnographic findings in children with laryngomalacia and other isolated larynx alterations, in other words, without alterations in their upper airways.

## MATERIALS AND METHODS

This work was carried out in the Mouth Breather Center - Otorhinolaryngology Department - Pediatric Otorhinolaryngology - Federal University of São Paulo, and the Sleep Institute from the Psychobiology Department of the Federal University of São Paulo. From 1999 to 2004, 29 children with laryngomalacia or other laryngeal alterations were selected. Their ages varied from 11 days of life to 8 years and 10 months. All of them underwent otorhinolaryngological exam, nasofibroscopy and polysomnography. We included only children with snoring and laryngeal stridor or vocal alteration in whom the fibroscopy exam showed laryngomalacia or other isolate laryngeal alteration. Laryngomalacia was considered when there was an excess of mucosal tissue in the posterior larynx wall, shortening of the aryepiglottic fold or collapse of epiglottis cartilage over the laryngeal lumen.

Children with adenotonsillar hypertrophy, craniofacial malformations or genetic syndromes were excluded.

Patients underwent day time polysomnography at the Sleep Institute - UNIFESP-EPM, where the exam was carried out with the patient sleeping in a comfortable bed, in a dark and quiet room. Electrophysiological and cardio-respiratory parameters were recorded in a computerized system (Alice®): electroencephalogram (4 channels), submentonian and tibial electromyogram, right and left electro-oculography, oronasal air flow, thorax and abdominal movements, microphone, oxyhemoglobin saturation (SaO2), and sleeping position. The patient was studied under regular room air. We plotted data related to age, nasofibroscopy and polysomnography. In order to analyze them, the children were divided in two groups: those with laryngomalacia and those with other laryngeal diseases (subglotic stenosis, hemangioma, synechiae, vocal fold paralysis, vocal gap, arytenoid fixation and/or vocal fold thickening).

In order to carry out a data statistical analysis, we used the Fisher Test, which did not show significance among the results.

All the patients handed in a duly signed informed consent form, and this research was approved by the Ethics Committee. (Protocol # 0122/02).

## RESULTS

Tables 1 and 2 depict the polysomnographic results found among the laryngomalacia patients and other laryngeal lesions, respectively.

**Table 1:** Polysomnography data observed in patients with laryngomalacia.

Patient	Age	naso	sat vig	SC	SA	NED	NEC	IAC	NEO	nadir	FC V	FC A	FC Q
TMS	6m	LM	98%	96%	93%	1	2	0.9	0	89%	167,5	135,1	135,6
LAB	5m	LM	95%	92%	92%	1	1	0.4	0	89%	145,0	123,5	126,0
ECM	5m	LM	90%	92%	93%	1	1	0.7	0	85%	173,0	151,4	158,8
GSS	6m	LM	96%	96%	96%	20	20	7.7	0	88%	163,2	139,1	134,2
DASS	9m	LM	98%	97%	98%	7	7	1.0	0	92%	138,2	126,9	119,8
SNR	5m	LM	95%	95%	95%	-	1	0.5	0	95%	indisp	indisp	indisp
TNB	2m	LM	96%	94%	96%	7	7	2.6	1	90%	174,9	136,9	141,0
SMS	11m	LM	92%	90%	89%	3	12	7.5	0	76%	158,7	141,9	138,4
PJS	3m	LM	98%	96%	95%	3	5	2.0	0	91%	160,2	130,1	134,0
MGF	6m	LM	97%	95%	93%	5	6	2.8	0	87%	indisp	indisp	indisp
LFSS	21d	LM	96%	96%	92%	6	8	4.2	0	91%	164,3	151,7	138,4
KMS	7m	LM	97%	97%	97%	10	12	4.6	0	88%	177,9	151,8	152,1
KSS	3m	LM	96%	96%	95%	4	18	6.9	0	92%	154,6	137,8	134,0
ENR	2m	LM	99%	98%	98%	3	18	8.9	0	93%	indisp	indisp	indisp
INJ	3m	LM	89%	93%	93%	-	2	1.0	0	90%	182,4	143,6	indisp
EGS	17m	LM	98%	95%	96%	-	24	2.6	7	92%	129,4	129,0	124,3
BNM	4m	LM	96%	96%	96%	1	2	1.5	0	92%	152,5	121,8	119,1
ASO	4m	LM	96%	94%	94%	13	30	25.0	1	86%	136,8	125,0	122,4

**Table 2:** Polysomnography data observed in patients with other laryngeal diseases.

Patient	Age	naso	sat vig	SC	SA	NED	NEC	IAC	IAH O	nadir
CRM	6a	bilateral vocal nodules	99%	98%	98%	indisp	0	0.4		92%
JCS	2a8m	Post muc fall glottis	99%	99%	99%	indisp	0	indisp		89%
PRC	8a	post arythenoid fix *	99%	97%	97%	indisp	0	indisp	0.8	95%
RAS	7a	Bilat VFs fixation*	98%	98%	98%	indisp	3	indisp		94%
SFC	7a	bilateral vocal nodules	98%	96%	96%	indisp	0	indisp	0.9	89%
KMF	13d	LVF idiopatic paralysis	98%	98%	97%	indisp	162	indisp		87%
MHS	1a	Obstant subglottic stenos 70%	96%	96%	97%	indisp	2	indisp		94%
OGST	1a	aryepiglothic fold shorten	93%	92%	92%	indisp	16	indisp		87%
AAA	6a	papilomatose obstr larynx	98%	96%	95%	indisp	0	indisp		92%
EPC	2a	arythenoid hemangioma	93%	92%	91%	indisp	0	indisp		72%
BGN	8a10m	subglottic stenosis	95%	95%	93%	indisp	0	indisp		84%

naso :nasofibroscopic diagnosis IAC: Central apnea index

Sat vig :saturation when awake NEO: number of obstructive events

SC :calm sleep nadir: lower oxyhemoglobin saturation

SA :active sleep FC V: heart rate when awake

NED :desaturation events number FC A: heart rate during active sleep

NEC :number of central events FC Q: heart rate at calm sleep

indisp :not available

Among the 18 children diagnosed with laryngomalacia, all of them had central-type respiratory events, and most of the episodes were related to oxygen desaturation and some to bradicardia. In this same group, 3 children had obstructive-type apnea. As to the 11 children with other laryngeal alterations, 4 had central-type respiratory events and 2 had obstructive type.

Oxygen saturation during sleep for these children varied from 76 to 98%.

Patient INJ, of three months of age has severe laryngomalacia, for he presented baseline oxygen saturation (awaken and during sleep) of 89%. Although the patient had 2 central-type respiratory events, there was an improvement in oxygen saturation during sleep.

## DISCUSSION

From 3 to 26% of the pediatric population seek specialized medical help because of snoring and mouth breathing, and only 1 to 3% of those have Sleep Obstructive Apnea Syndrome (SOAS). This disease is suspected through history taking; however this alone is unable to separate primary snorers from SOAS patients. Polysomnography is indicated in these cases, because besides defining diagnosis, it also characterizes the type of event that happened, obstructive, central or mixed, and also assessing disease severity through the Hypopnea/apnea index (HAI) and oxygen saturation level. It is important to remember that, as far as physiopathology and polysomnographic diagnostic criteria are concerned, SOAS in children is considered a different disease from the one that affects adults.

Otorhinolaryngological exam, together with nasofibroscopy is able to find most of the apnea causes.

Although most of the times it is caused by adenotonsillar hypertrophy, we must bear other causes in mind, laryngeal diseases amongst them.

Laryngomalacia physiopathology is still controversial. Among the most accepted theories we have the immaturity of the nervous system. This finding is in agreement with what has been seen in the paper by Milczuk & Johnson^29^, in which children with laryngomalacia presented comorbidities such as LFR, prematurity and DNPM delays, amongst others, all of them related to some degree of neurological immaturity.

Short duration central apnea is considered a normal finding during the sleep of newborns and, so far, there is no criterion that defines an acceptable number of central apneas. This type of apnea, in order to be considered relevant, should last more than two respiratory cycles, or be associated to bradicardia and/or cyanosis. Among the 18 children diagnosed as having laryngomalacia, 18 had central-type respiratory events, and most of those episodes were associated to oxygen desaturation and some to bradicardia. In this same group, 3 children had obstructive-type apnea. As to the 11 children with other laryngeal alterations, 4 had central-type respiratory events and 2 had the obstructive type.

In general, the number of events was related to the desaturation severity. However, two cases differed from the majority: we may mention ENR, that despite having 18 respiratory events, none of them were associated to oxygen desaturation below 93%, while OGST had practically the same number of events, however with relevant oxygen desaturation (87%).

## CONCLUSION

Most of the children with laryngomalacia or other laryngeal diseases did not have significant respiratory alterations during sleep. However the group of infants with laryngomalacia presented more central-type apneas. In this group, although most of the respiratory events may be considered benign, their greater frequence suggests partial obstruction of the upper airways.

Having seen the findings of our study, we believe the evaluation of respiratory disorders during sleep in patients with laryngeal disorders, using modern methods provided with pressure sensors to detect nasal air flow, may bring new data for the understanding of the physiopathology of these diseases, and this is what we intend to do in a continuation of the present investigation.
